# CRISPR/Cas-mediated germplasm improvement and new strategies for crop protection

**DOI:** 10.1007/s44297-023-00020-x

**Published:** 2024-02-01

**Authors:** Ganggang Dong, Zaifeng Fan

**Affiliations:** 1 Sanya Institute of China Agricultural University, Sanya, 572025 China; 2 MARA-Key Laboratory of Surveillance and Management for Plant Quarantine Pests, College of Plant Protection, China Agricultural University, Beijing, 100193 People’s Republic of China

**Keywords:** Genome editing, CRISPR/Cas, Germplasm improvement, Crop protection, Global regulations

## Abstract

Global agriculture and food security are encountering unprecedented challenges from both the ever-growing population and rapidly changing climate conditions. CRISPR/Cas-mediated genome editing technology has revolutionized plant functional genetic research and precision crop breeding with robustness, high target specificity and programmability. Furthermore, numerous emerging biotechnologies based on the CRISPR/Cas platform provide the opportunity to create new crop germplasms with durable resistance against disease or insect pests, herbicide tolerance, and other stress-tolerant improvements, reshaping crop protection to increase agricultural resilience and sustainability. In this review, we briefly describe the CRISPR/Cas toolbox, including base editing, prime editing, compact genome manipulation, transcriptional regulation and epigenetic editing, and then overview the most important applications of CRISPR/Cas-mediated crop genetic improvement, highlighting crop protection-based stress resistance engineering. In addition, we enumerate global regulations on genome-edited crops. Finally, we discuss some bottlenecks facing this cutting-edge technology and infinite possibilities for the future.

## Introduction

The global population is rapidly increasing and estimated to reach ca. 10 billion by 2050 [1], posing unprecedented challenges to agriculture and food security amidst accelerating climate change. Factors like global warming, shrinking arable land, groundwater depletion, restrictions on pesticides and chemical fertilizers, and the requirement for carbon neutrality and benchmarking have impacted crop production, which appears to be plateauing or even declining. Furthermore, the prevalence of diseases, arthropod pests, and weeds threatens plant growth, while the lack of durable, broad-spectrum resistant crop varieties aggravates production issues. Conventional hybridization and mutation breeding based on natural or induced genetic polymorphisms are labor intensive and time consuming. Therefore, to feed a population of 10 billion, existing crop yields must increase by 60% in the near future [2]. Innovative crop improvement strategies like genome editing can be employed to boost agricultural productivity and sustainability.

Functional genetic variation and diversity are crucial for agricultural improvement. Advances in plant genome engineering and sequencing technologies have upgraded crop breeding from cross- and mutation-breeding to transgenic- and gene editing-breeding [3, 4]. Transgenic breeding introduces desirable traits into elite cultivars by inserting exogenous genes, circumventing reproductive barriers. These technologies have to some extent increased crop disease resistance, reduced pesticide usage, improved nutrition, higher yields and quality, and greater environmental resilience and facilitated the creation of new germplasms and the breeding of new varieties. However, genetically modified crops face many bottlenecks due to concerns over off-target effects, uncertainties about exogenous gene integration, and public apprehensions regarding biosafety. Loss-of-function gene mutants and gain-of-function germplasms are critical resources for understanding gene function and crop genetics improvements, specifically regarding plant susceptibility and resistance. Genome-editing technologies have dramatically expanded the capabilities of sequence-specific nucleases (SSNs), enabling the precise identification, localization, and modification of specific genomic sequences [5]. Four major classes of nucleases i.e., meganucleases, zinc finger nucleases (ZFNs), transcription activator-like effector-based nucleases (TALENs), and CRISPR (clustered regularly interspaced short palindromic repeats)-Cas (CRISPR-associated protein), have been successfully employed for plant and animal genome editing [6]. The CRISPR/Cas system is one of the most advanced and innovative plant genome-editing systems, providing targeted modification of functional genes and allowing for precise breeding with high specificity and programmability. It has been successfully applied for genome engineering in various eukaryotes, including rice (*Oryza sativa*), wheat (*Triticum aestivum*), maize (*Zea mays*), potato (*Solanum tuberosum*) and cassava (*Manihot esculenta*) [7, 8], particularly in plant‒pathogen interaction studies covering fungi, bacteria, viruses and oomycetes, among many others [9]. Moreover, several emerging technologies derived from the CRISPR/Cas-platform boosted the development of basic research and synthetic biology [8].

In this review, we overview the features and mechanisms of CRISPR/Cas-based genome editing technology, discussing its innovative applications, such as base editing and prime editing, and exploring advancements in reagent delivery methods, gene regulation, multiplexed gene editing and epigenome editing. Furthermore, we summarize recent progress in utilizing CRISPR/Cas-mediated genome editing in crop breeding for disease resistance, pest control, and herbicide tolerance in major crops like maize, rice, wheat and potato. Finally, we emphasize the significance of effective regulation for CRISPR/Cas-related technologies to ensure responsible development and adoption of edited crops and their derivatives in different countries.

## Evolving CRISPR/Cas systems: the basic machinery and large family

CRISPR‒Cas, an RNA-mediated bacterial adaptive immune system, is divided into two major classes [10]. The class 1 system requires multiple Cas proteins to form a functional complex and cleave target DNA sequences, while the class 2 system only needs a single, larger Cas protein [11]. The CRISPR systems are subdivided into six types and 33 subtypes based on their modes of cleavage and activity on DNA/RNA substrates [12]. The class 1 system comprises types I, III, and IV, while the class 2 system includes types II, V, and VI [13, 14]. Types I, II, and V systems target DNA genomes, while types II, IV, and VI systems can also modify RNA substrates [15]. The class 2 system is widely used in genome editing, and designing and delivering a gRNA is relatively straightforward and effective.

### The CRISPR/Cas9 system

**Fig. 1:**
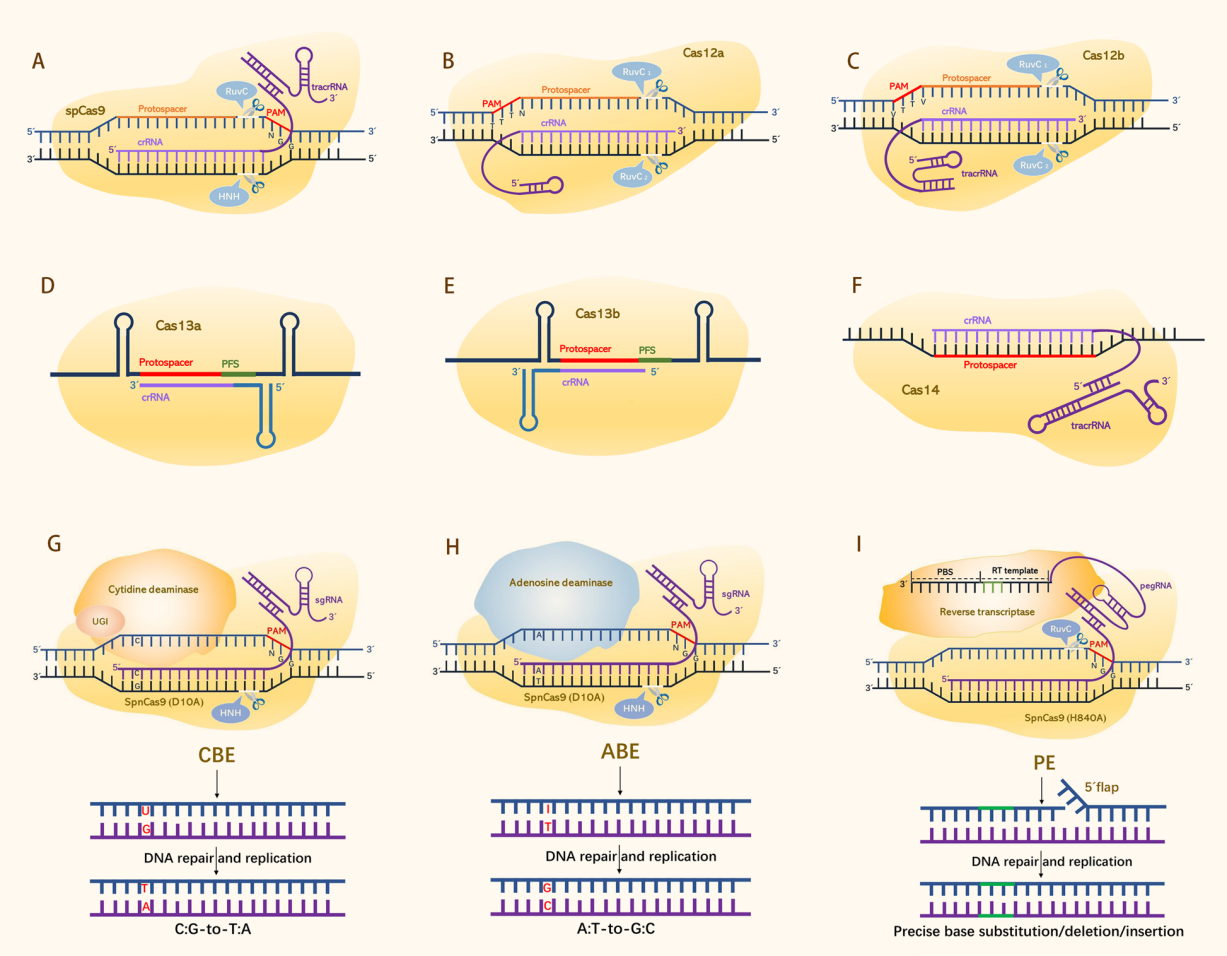
CRISPR/Cas systems used for genome editing in plants. Notes: **A** The CRISPR/Cas9 system comprises the SpCas9 endonuclease with two catalytic domains (RuvC and HNH) and a sgRNA containing crRNA and tracrRNA. The sgRNA directs the complex to a specific DNA locus located upstream of a GC-rich PAM sequence (5´-NGG-3´), creating DSBs with blunt and/or staggered ends upon target DNA cleavage. **B** The CRISPR/Cas12a system comprises the Cas12a endonuclease, guided by a single mature crRNA, which binds to the target DNA locus downstream of a T-rich PAM (5´-TTTN-3´) sequence, resulting in DSBs with staggered ends via a singular RuvC catalytic nuclease domain after certain conformational transitions. **C** The CRISPR‒Cas12b system relies on the Cas12b endonuclease, which carries a single RuvC catalytic domain responsible for mediating staggered DNA cleavage, along with a sgRNA (including crRNA and tracrRNA) guiding the complex to a specific site downstream of a T-rich PAM (5´-VTTV-3´). **D, E** The CRISPR/Cas13 system includes a Cas13 protein featuring two distinct HEPN RNase domains. The crRNA-Cas13 complex identifies a 5´-and/or 3´-protospacer-flanking site (PFS) sequence and cleaves ssRNA targets guided by gRNA, independent of the PAM. **F** The CRISPR/Cas14 system utilizes a Cas14 ribonucleoprotein with a conserved RuvC domain to target and cis-cleave ssDNA guided by gRNA, without the strict requirement for a PAM sequence. **G** Cytosine base editors (CBEs) are composed of nCas9 (D10A) fused to a cytidine deaminase catalytic domain (such as rAPOBEC1, PmCDA1, hAID, or hA3A), in conjunction with uracil DNA glycosylase inhibitors (UGIs), coordinating the C: G > T: A base substitution in the designated “editing window” at the 5´ end of the non-targeted sequence. **H** Adenine base editors (ABEs) are composed of nCas9 (D10A) fused with an adenosine deaminase catalytic domain (ecTadA-ecTadA*), allowing for an A:T > G:C base exchange within the specified “editing window” at the 5´ end of the non-targeted sequence. **I** Prime editors (PEs) are composed of nCas9 (H840A) fused with a reverse transcriptase (RT) and a pegRNA, allowing insertions, deletions, and all kinds of base substitutions. nCas9 induces a break in the PAM-containing DNA strand, and the primer-binding site (PBS) sequence initiates reverse transcription by hybridizing with the cleaved ssDNA upstream of the break. This process leads to the accurate incorporation of edited nucleotides into the desired DNA sequence. The models depicted are not to scale and solely serve for illustrative representation

The leading CRISPR/SpCas9 system, a type II class 2 genome editor from *Streptococcus pyogenes*, includes a single plasmid encoding the DNA endonuclease SpCas9, and an engineered single-guide RNA (sgRNA) by artificially fusing a small mature CRISPR RNA (crRNA) and a *trans-*activating crRNA (tracrRNA) [16, 17]. The system is activated when Cas9 binds sgRNA, allowing the sgRNA to recognize a GC-rich (5´-NGG-3´) protospacer adjacent motif (PAM) and guide Cas9 to the designated DNA site, inducing a blunt-ended DNA double-strand break (DSB) at the 3-bp position upstream of the PAM (Fig. 1A) [18]. Non-homologous end joining (NHEJ) and homology-directed repair (HDR) are two cellular DNA repair pathways, among which NHEJ is used to repair DSBs and introduce indel genetic variations in the CRISPR/Cas9 editing case [19]. The CRISPR/Cas9 system is now routinely used in numerous plants. One of the most significant limitations is the large size of Cas9 and the requirement of the PAM sequence for DNA target cleavage. Research efforts are needed to improve its accuracy and serviceability by creating Cas variants, e.g., SpCas9-NG, SpCas9-NRRH, SpCas9-NRCH, SpCas9 (VQR), SpG, and SpRY [20, 21]. Recently, Yin et al*.* developed a new Cas9 variant, Cas9TX, which optimizes both SpCas9 and TREX2 to suppress chromosomal translocation levels, increasing the safety and efficiency of single-site editing. This approach mitigates perfect DNA repair and excessive cleavage risks, presenting a viable solution to extant challenges [22].

### The CRISPR/Cas12 systems

The second leading genome-editing tool, the class 2 type V-A CRISPR/Cas12a system, or Cpf1, is emerging as a promising alternative to CRISPR/Cas9. Composed of ca. 1200 amino acids (aa), it employs a single mature crRNA for DNA target cleavage, producing staggered ends [23]. Cas12b, or C2c1, a type V-B system derived from *Alicyclobacillus* spp., is smaller (only 1129 aa) and requires both tracrRNA and crRNA for targeting specificity [24]. Cas12a and Cas12b recognize an AT-rich PAM (5´-TTN for FnCas12a) and generate 4- to 5-nt staggered ends distal to the PAM (Fig. 1B & C) [25]. Ming et al*.* developed AaCas12b, AacCas12b, and BthCas12b editing systems for rice, with AaCas12b demonstrating superior efficiency in multiplexed genome editing [26]. Zegeye et al*.* reported an optimized Cas12a variant, enAsCas12a, expanding the targeting range beyond the canonical TTTV PAMs, allowing for a wider range of DNA modifications [27]. To enrich the plant genome editing toolbox, endeavors have been undertaken to explore the CRISPR/Cas12a system, which can provide robust genome editing efficiency at lower temperatures. Li et al*.* scrutinized 17 novel Cas12a orthologs for their genome editing capabilities in plants. Among them, Ev1Cas12a and Hs1Cas12a demonstrated effective multiplexed genome editing capability in rice and tomato protoplasts, indicating a promising new genome-editing tool [28]. Furthermore, applications such as CRISPR/Cas12-mediated DETECTR for pathogen diagnostics and Cas12a coupled with loop-mediated isothermal amplification (LAMP) for rapid detection of plant viruses highlight the versatility of this system [29, 30].

### The CRISPR/Cas14 system

The class 2 type V CRISPR/Cas14 system, which originated from archaea, consistently exhibits high specificity and efficiency in single-stranded DNA (ssDNA) cleavage without the PAM sequence requirement (Fig. 1F). This characteristic can facilitate robust defense against ssDNA viruses in crops and enable high-fidelity SNP genotyping [31, 32]. With a length of ca. 400–700 aa (40–70 kDa), Cas14 has 24 variants belonging to the subgroups Cas14a, Cas14b, and Cas14c [31]. Additionally, Cas14 holds potential for precise diagnostics of plant viruses and bacteria, as well as early detection of human cancer cells [32].

### The CRISPR/Cas13-mediated RNA editing system

CRISPR/Cas13, a class 2 type VI system, is subdivided into four subtypes: VI-A (Cas13a), VI-B (Cas13b), VI-C (Cas13c), and VI-D (Cas13d) [33, 34]. Cas13 possesses two distinct higher eukaryotes and prokaryotes nucleotide-binding (HEPN) RNase domains that require a crRNA-guided protein complex to redirect the RNA site through a 5´- and/or 3´-protospacer flanking sequence (PFS) (Fig. 1D & E) [35]. The Cas13 system, functioning as an RNA-guided ribonuclease, is an indispensable tool in the realm of RNA manipulation and transcriptional regulation due to its programmability and specificity. Nonetheless, the large size of Cas13 effectors (967–1152 amino acids) and the inherent non-specific RNA cleavage upon target activation have limited the potential for packaging into adeno-associated viruses for subsequent in vivo delivery in therapeutic contexts. Deng et al*.* reported a compact Cas13 (Cas13bt3), which exhibits compatibility for adeno-associated virus delivery. Notably, it selectively cleaves both targeted RNA and non-specific RNA at internal "UC" sites and is activated in a target length-dependent manner. Additionally, they engineered a Cas13bt3 variant with minimal off-target cleavage while preserving its efficacious target cleavage capabilities [36]. The CRISPR/Cas13 system, including specific high-sensitivity enzymatic reporter unlocking (SHERLOCK), has become popular for detecting plant pathogens, showing significant practical potential [37]. Recently, Cas13-independent guide-induced gene silencing (GIGS) employed targeted crRNA without Cas13 protein to substantially reduce viral and endogenous RNA levels in tobacco, tomato, and *Arabidopsis*. GIGS offers a powerful biotechnological platform, especially for tissue- or time-specific expression that is difficult to manipulate precisely with CRISPR/Cas9 through the regulation of pleiotropic regulatory genes [38].

### CRISPR/Cas-mediated compact genome manipulation

The large size of Cas9 and Cas12 limits their flexibility in gene editing and therapeutic applications. Compact Cas nucleases are needed to overcome cargo size limitations in effective delivery vehicles like adeno-associated virus (AAV). CRISPR‒CasX, or Cas12e, is a compact protein belonging to the class 2 family, with a size below 1,000 aa, which possesses unique domains distinct from Cas9 and Cas12 but shares the RuvC nuclease domain with less than 16% identity. As an RNA-guided DNA endonuclease, CasX efficiently generates staggered DSBs at a sequence complementary to the 20 nucleotides within its guide RNA [39]. CRISPR‒CasΦ (Cas12j) is a hypercompact genome editor found in huge bacteriophages, comprising a single 70-kDa CasΦ protein and a CRISPR array. Despite its molecular mass being half of Cas9 and Cas12, it can generate mature crRNA and cleave target exogenous DNA via a single active site. Cas12j offers advantages in genome editing, DNA detection, and cellular delivery and has been successfully applied to human and plant cells [40]. CRISPR-AsCas12f1 is another miniature class 2-type V-F system consisting of only 422 aa. It is an RNA-guided endonuclease that recognizes 5´ T-rich PAMs and creates staggered DSBs in DNA for cellular delivery and programmable genome editing in bacterial and mammalian cells [41]. Recently, Hino et al*.* upgraded the ultra-miniature AsCas12f system through structural analysis, protein engineering, and sgRNA optimization, resulting in the creation of two AsCas12f activity-enhanced (enAsCas12f) variants. These variants exhibit genome editing activity in human cells comparable to SpCas9 and AsCas12a. The engineered enAsCas12f shows high effective gene knockout and insertion editing efficiency in cellular and animal models and efficient gene activation capability, with substantial advantages in terms of "body size", highlighting its significant potential for clinical therapeutic applications [42]. The CRISPR/Cas system is a well-known ancient immune system originating from prokaryotes (bacteria and archaea) that is used to protect against the invasion of foreign genetic elements. While CRISPR systems have been discovered in viruses (bacteriophages), it remains elusive whether a similar system exists in eukaryotes. Altae-Tran et al*.* reported a new class of prokaryotic RNA-guided systems termed OMEGA, which includes lscB, lsrB and Tnp8. The OMEGA effector TnpB is the putative ancestor of Cas12, suggesting that TnpB may also be the ancestor of the Fanzor (Fz) protein encoded by eukaryotic transposons [43]. Recent research indicates that the Fanzor protein from eukaryotes is an RNA-guided DNA endonuclease [44]. Compared with the CRISPR/Cas system, the Fanzor system is more compact and exhibits collateral activity, making it easier to be delivered into cells for more precise genome editing. This study suggests that RNA-guided endonucleases exist in all three domains of living organisms [44].

### CRISPR/Cas-derived base editors

CRISPR/Cas-derived base editors (BE) provide powerful and DSB-free tools for programmable single DNA base substitution in plants. Cytidine/cytosine base editors (CBEs) and adenine base editors (ABEs) are fusion proteins composed of catalytically impaired Cas9 (nCas9/dCas9) and cytidine deaminase (CD) or adenine deaminase (AD). These base editors recognize single-stranded DNA (ssDNA) sequences guided by gRNAs, and introduce precise cytosine-to-thymine (C-G to T-A) or adenine-to-guanine (A-T to G-C) base substitutions without cleaving the DNA in an “R-loop”, respectively (Fig. 1G & H) [45, 46]. Popular BE systems include BE3, BE4, targeted AID and dCpf1-BE [47–50]. PmCDA1 and rAPOBEC1 are commonly used cytosine deaminases for plant genomes, which can catalyze C-G to T-A or A-T to G-C transitions in *Arabidopsis*, tomato, and potato, albeit with a large number of indels [51]. CBEs have been used in maize and wheat to induce herbicide resistance by specific base substitutions in genes such as *OsALS1* and *OsACC* [52]. However, single-base editing systems have a limited editing window [53]. Dual base editors combine cytidine and adenine deaminases to induce simultaneous C-G to T-A and A-T to G-C mutations at the same editing site, expanding the range of editable bases in plants [54]. Additionally, traditional BEs lead to unpredictable guide RNA (gRNA)-independent off-target editing in the genome and transcriptome due to the spurious activity of BE-enclosing deaminases. Existing optimization methods only focus on deaminase-specific mutations and the use of exogenous regulators. Xiong et al*.* developed a system of split deaminase for safe editing (SAFE), which ensures the correct splitting of the deaminase domain embedded in a Cas9 nickase. This process simultaneously fragments and inactivates the deaminase and Cas9 nickase, providing a robust target-editing ability in plants, human, and yeast cells, while minimizing both gRNA-independent and gRNA-dependent off-target DNA/RNA edits. SAFE offers a generalizable solution for BEs that do not require external regulators [55]. These editing tools offer great potential for precise manipulation of genes governing critical agronomic traits.

### CRISPR/Cas-derived prime editing

CRISPR-mediated base editors provide precise base transversions in eukaryotic genomes. However, it remains challenging to inducing predictable insertions and deletions. Prime editing technology allows all twelve types of base substitutions, targeted insertions, and deletion editing in human cells without the need for DNA donor templates or inducing DSBs [56, 57]. Prime editors (PEs) are multifunctional tools that involve a fusion protein comprising engineered nCas9 with an impaired HNH domain (H840A) and reverse transcriptase (RT). This fusion protein is assembled with an engineered prime editing guide RNA (pegRNA) that comes with 3´-extended bases, containing both a primer binding site (PBS) and an RT template coding for the desired edits, specifically guiding the editing protein complex [58]. Once the nCas9 component of the complex identifies and cleaves the target site, it releases a PBS-paired site-specific ssDNA break as a primer for RT. This ssDNA strand then pairs with the PBS, initiating reverse transcription, transferring the new genetic information encoded by pegRNA to the non-target DNA strand, and precisely integrating the edits into the target site via DNA repair mechanisms, enabling highly accurate editing (Fig. 1I) [58]. Plant prime editors (PPEs) have been used to enhance disease resistance and stress tolerance in wheat, rice, and *Arabidopsis* [59].

### CRISPR/Cas-mediated transcriptional regulation and epigenetic editing in plants

**Fig. 2:**
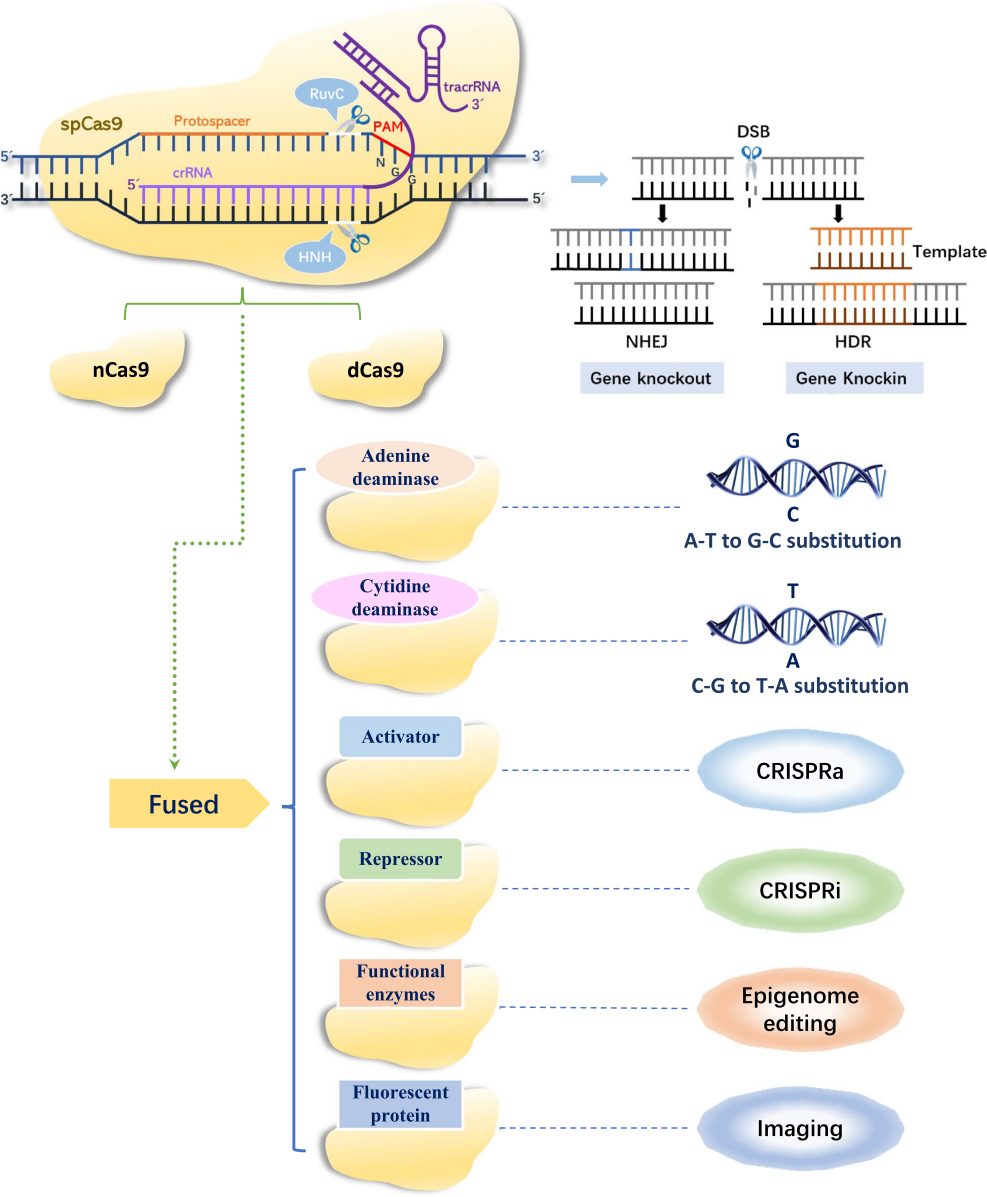
The CRISPR/Cas system used for the functional research of plant genes (modified from Zhang et al*.* [5]). Notes: The Cas protein contains a DNA binding domain and nuclease activity domains. The CRISPR/Cas system is widely utilized for gene knockout or silencing of individual genes by triggering the cell's NHEJ repair mechanisms through the insertion or deletion of one or more nucleotides. Additionally, it can be used for gene knock-in, over-expression of individual genes, or replacement of undesirable genes through the use of template DNA that requires HDR. Furthermore, with the incorporation of engineered enzymes, such as nickase Cas9 (nCas9), deactivated Cas9 (dCas9) fused with base conversion enzymes (cytidine deaminase, CD and adenine deaminase, AD), transcription effectors (such as activators or repressors), and other enzymes (methyltransferases, demethylases, acetylases), as well as fluorescent proteins, the applications of the CRISPR/Cas system are becoming increasingly versatile. It can be employed for precise base editing, transcriptional modulation (CRISPRa or CRISPRi), programmed epigenetic editing (including DNA methylation, histone modification, and non-coding RNAs), and real-time live imaging for genomic loci and transcript mobility [5]

CRISPR/Cas-mediated transcriptional regulation involves inactivated Cas proteins (dCas), which have mutations that disable catalytic DSB cleavage functions but retain DNA recognition activity with the gRNA. In the CRISPR/dCas interference (CRISPRi) system, the dCas protein specifically binds to the promoter transcription start site (TSS) region or the coding region and fuses with transcriptional inhibitors, like Krüppel-associated box domain of Kox1 (KRAB) or repression domain of ERF transcription factor (SRDX), to disrupt transcription factor and RNA polymerase (RNAP) transcription initiation or elongation pathways to regulate gene expression [60]. In addition, the CRISPR/Cas-derived transcription activation (CRISPRa) system increases endogenous gene transcription and activates multiple genes by fusing engineered dCas9 proteins with transcriptional activators, such as VP16 (herpes simplex Viral Protein 16), tetrameric repeat VP64, TAL (transcription activator-like), EDLL (activation domain of TDR1 transcription factor), and ethylene responsive factor/ethylene-responsive element binding proteins (ERF/EREBP), or using scaffold RNA (scRNA) to recruit them [61]. In theory, the dCas enzyme, along with multiple bio-regulatory components, can be used to target transcriptional regulation, programmed epigenetic editing (such as DNA methylation, histone modification, and non-coding RNAs), real-time, live imaging of chromatin and gene localization (Fig. 2). The CRISPRa system has evolved from the dCas9-VP64-based CRISPR-Act1.0 to more potent CRISPR-Act2.0 (namely, dCas9-SunTag, dCas9-TV, and dCasEV2.1), and multiplexed gene activation CRISPR-Act3.0 [62–65]. Pan et al*.* recently developed the CRISPR-Combo platform to enable simultaneous genome editing (targeted mutagenesis or base editing) and gene activation in several plants, including *Arabidopsis*, *Populus*, and rice, using two sgRNAs, gR1.0 and gR2.0, with 20-nt and 15-nt protospacers, respectively [66]. CRISPRoff is a programmable epigenetic editor that uses a single dCas9 fusion protein to enable durable, heritable, and reversible DNA methylation modifications and gene transcriptional regulation, independent of the classic promoter CpG islands (CGIs) structure, providing diverse applications in genome-wide screens, multiplexed cell engineering, enhancer silencing, and exploring epigenetic inheritance mechanisms [67]

## Applications of CRISPR/Cas-based genome editing in crop improvement

**Fig. 3:**
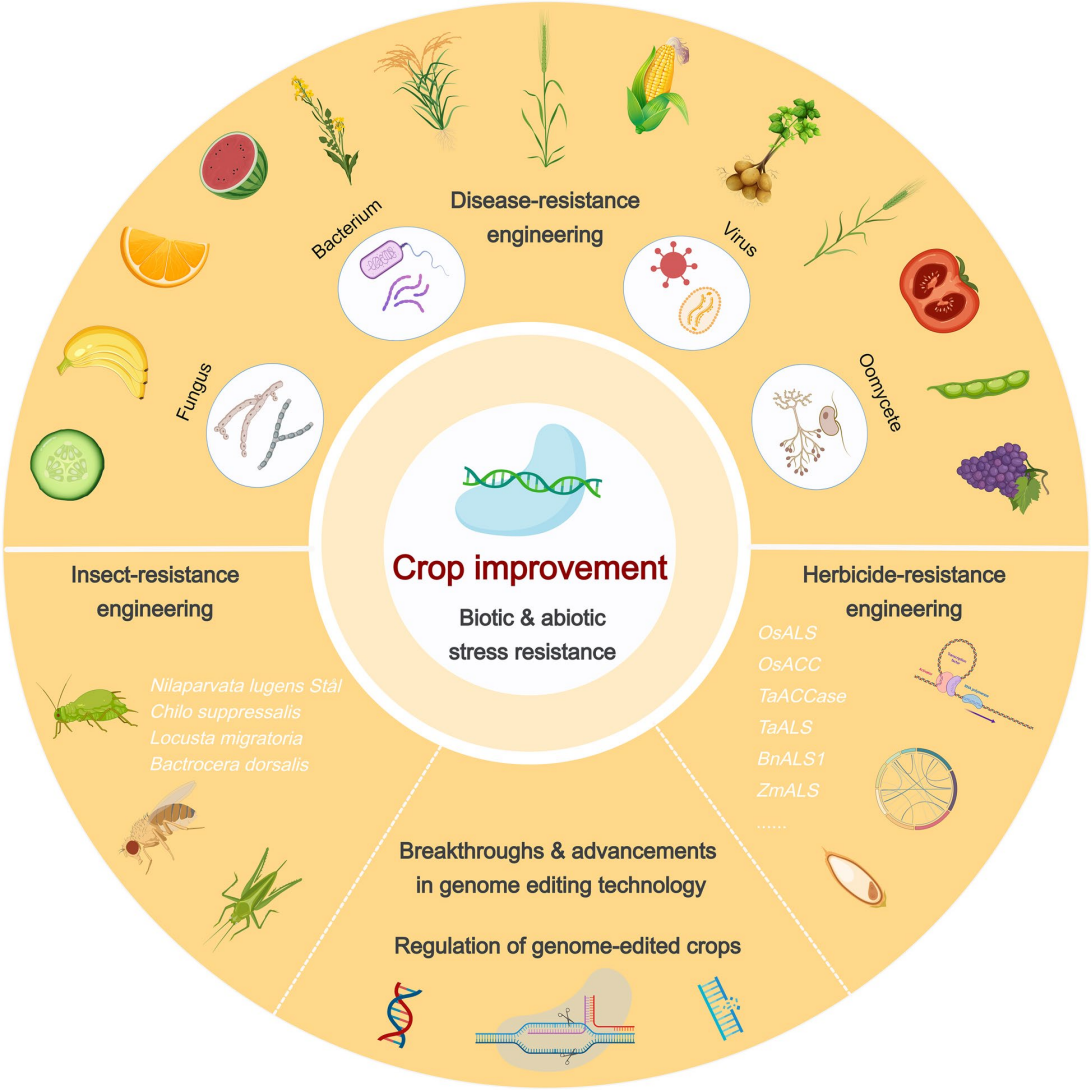
Graphical abstract. CRISPR/Cas-mediated crop improvement engineering

CRISPR/Cas-mediated genome editing technology has demonstrated exceptional efficiency and productivity in crop trait improvement, making remarkable contributions to improving key agronomic traits of crops, including enhanced resistance to various fungal, bacterial, oomycetes and viruses, insect resistance, and herbicide tolerance. These advancements have generated abundant novel genetic materials, which has accelerated the selection and breeding of high-quality crop varieties, further promoting the development of new crop protection engineering (Fig. 3). In the following sections, we will review the latest research and applications of CRISPR/Cas-based editing systems, especially focusing on improving staple crops like rice, wheat, maize, and potato, as well as some other crucial crops (Table 1).

**Table 1:** CRISPR/Cas-mediated disease resistance for crop improvement

Crop	Pathogen	Targeted gene	Results	CRISPR/Cas	Mutation type	Reference
Wheat	Fungus	*TaMLOs*	Resistance to *Blumeria graminis* f.sp. *tritici* (*Bgt*)	CRISPR/Cas9	knockout loss-of-function mutation	[68]
		*TaMLO-R32*				[69]
		*TaEDR1*				[70]
		*TaWRKY19*	Resistance to *Puccinia striiformis* f.sp. *tritici* (*Pst*)			[71]
		*TaPsIPK1*				[72]
		*TaHRC*	Resistance to *Fusarium verticillioides* &* F. graminearum*			[73]
Rice	Fungus	*OsERF922*	Resistance to *Magnaporthe oryzae*			[74]
		*OsTGA5*				[75]
		*OsSEC3A*	Varying degrees of resistance to *Magnaporthe oryzae*			[76]
		*Pita, Pi21*				[77]
		*Bsr-k1, Bsr-d1*				[78]
		*RBL1*	Resistance to *Magnaporthe oryzae*			[79]
			Resistance to *Ustilaginoidea virens*			
	Bacterium	*OSWEET11,13,14*	Resistance to *Xanthomonas oryzae* pv*. oryzae* (*Xoo*)			[80]
		*OsEDR1*				[81]
		*Xa13*				[82]
		*Pong2-1,11–1*				[83]
		*Os8N3*				[84]
		*RBL1*				[79]
		*Xig1*				[85]
		*OsSULTR3;6*	Resistance to *Xanthomonas oryzae* pv*. oryzicola* (*Xoc*)			[86]
	Virus	*elF4E*	Resistance to rice tungro spherical virus (RTSV)			[87]
Maize	Fungus	*ZmFER1*	Resistance to *Fusarium verticillioides* &* F. graminearum*		deletion/insertion	[88]
		*ZmLOX3*	Resistance to smut (*Ustilago maydis*)		frame shift mutation	[89]
		*ZmCOIa*	Immunity to Gibberella stalk rot (*F. graminearum*)			[90]
		*ZmJAZ15*	Resistance to Gibberella stalk rot (*F. graminearum*)		knockout	[90]
Potato	Oomycete	*StDMR6-1*	Resistance to *Phytophthora infestans*			[91]
Barley	Fungus	*MORC1, MORC6a*	Resistance to *Blumeria graminis* &* F. graminearum*		double knockout	[92]
	Virus	*PDIL5-1*	Resistance to barely mild mosaic virus (BaMMV)		knockout	[93]
Tomato	Fungus	*SIMLO1*	Resistance to *Oidium neolycopersici*			[94]
	Bacterium	*SIJAZ2*	Resistance to *Pseudomonas syringae* pv. *tomato* DC3000			[95]
	Bacterium Oomycete	*SIDMR6-1*	Resistance *Xanthomonas* spp. *Phytophthora syringae* &* Phytophthora capsica*			[96]
	Virus	*elF4E*	Resistance to pepper mottle virus (PepMoV)			[97]
Soybean	Fungus	*GmMLO*	Resistance to *Erysiphe diffusa* (Cooke & Peck)			[98]
	Oomycete	*GmTAP1*	Resistance to *Phytophthora sojae*		deactivate	[99]
Grapevine	Fungus	*VvMLO3/4*	Resistance to *Erysiphe necator*		deletion/insertion	[100]
Rapeseed	Fungus	*BnQCR8*	Resistance to *Sclerotinia sclerotiorum* &* Botrytis cinerea*		knockout	[101]
Watermelon	Fungus	*Clpsk1*	Resistance to *Fusarium oxysporum* f.sp. *niveum* (*FON*)			[102]
Citrus	Bacterium	*CsLOB1*	Resistance to *Xanthomonas campestris* pv*. citri* (*Xcc*)			[103]
						[104]
		*CsWRKY22*				[105]
Banana	Bacterium	*MusaDMR6*	Resistance to *X. campestris* pv. *musacearum* (*Xcm*)			[106]
Cucumber	Virus	*elF4E*	Resistance to zucchini yellow mosaic virus (ZYMV) cucumber vein yellowing virus (CVYV) papaya ringspot mosaic virus-W (PRSMV-W)			[107]
Cassava	Virus	*elF4E*	Resistance to cassava brown streak virus (CBSV) Ugandan cassava brown streak virus (UCBSV)			[108]

### CRISPR/Cas-mediated pathogen resistance engineering

The response of host plants to pathogen infection involves a complex interplay of multiple genes. Genome editing has emerged as an effective approach to regulate specific defense mechanisms and enhance plant disease resistance by modifying genes for immune NLR receptors, resistance (*R*) or susceptibility (*S*) and targeting the degradation of the viral genome. In plant-microorganism interactions, both *S* genes and negative regulators of plant innate immune responses are favorable targets for CRISPR/Cas-based gene editing to strengthen plant disease resistance. Interfering with host susceptibility factors using the CRISPR/Cas toolbox is considered more practical and effective than the expression of *R* genes to promote pathogen resistance and protect plants from biotic stresses [109]. Consequently, a CRISPR/spCas9-mediated genome editing system has been successfully established for resistance to various plant pathogens, particularly fungal diseases.

### Resistance against fungal pathogens

Powdery mildew is a prevalent fungal disease that causes severe damage to many crops, like wheat, tomato, and strawberry. Mildew resistance locus O (*MLO*) is a well-studied *S* gene found in barley. Inhibiting its expression can enhance resistance to barley powdery mildew. Wang et al*.* employed CRISPR/SpCas9 to knockout the *TaMLO* gene in hexaploid bread wheat, establishing durable and broad-spectrum resistance to powdery mildew caused by *Blumeria graminis* f.sp. *tritici* [68]. However, *MLO*-associated resistance exhibits some negative phenotypes, such as early senescence, growth inhibition, and yield losses. Li et al*.* screened a novel *MLO* mutant (*TaMLO-R32*) with a targeted deletion of 304 kb in the *MLO-B1* locus of wheat. Along with alterations in the three-dimensional structure of the chromosome, leading to overexpression of the upstream gene tonoplast monosaccharide transporter 3 (*TaTMT3B*) is overexpressed [69]. This activation alleviates negative phenotypes caused by *MLO* disruption, effectively achieving a win‒win scenario for high crop yields and resistance. Moreover, the study employed CRISPR/Cas9 to create new wheat germplasms harboring precise mutations of the *TaMLO-R32* allele with broad-spectrum resistance to powdery mildew, covering the main wheat varieties [69]. Similarly, CRISPR/Cas9-mediated targeted mutagenesis of the *SIMLO1* gene in tomato and the *VvMLO3/4* gene (homolog of *Arabidopsis AtMLO2/6/12* gene) in grapevine has generated transgene-free tomato materials and four *VvMLO3*-edited lines that are resistant to powdery mildew [94, 100]. Additionally, Bui et al*.* employed a dual-gRNA CRISPR/Cas9 system to induce targeted mutations in the soybean *GmMLO* gene, successfully knocking out four homologs—*GmMLO02*, *GmMLO19*, *GmMLO20*, and *GmMLO23*—simultaneously in the soybean elite cultivar DT26. The *Gmmlo* mutant lines exhibited increased resistance to soybean powdery mildew and showed no significant differences in morphology, development, and productivity compared with the wild type [98]. Furthermore, enhanced disease resistance 1 (*EDR1*) encodes a MAPK kinase that negatively regulates host defense responses to powdery mildew. Loss-of-function mutations of the *EDR1* gene in *Arabidopsis thaliana* enhanced programmed cell death under multifarious biotic and abiotic stresses [110]. Simultaneous disruption of three wheat homologs of *TaEDR1* genes using CRISPR/Cas9 resulted in significant resistance to powdery mildew caused by *B. graminis* f.sp. *tritici* [70].

Wheat stripe rust, caused by *Puccinia striiformis* f.sp. *tritici* (*Pst*), is a destructive airborne fungal disease. Conventional breeding, which relies on a single *R* gene, is often overcome by persistently evolving pathogens. Mutations or deletions in *S* genes offer a promising alternative for acquiring durable broad-spectrum resistance. Wang et al*.* identified the *S* gene *TaWRKY19*, which acts as a transcriptional repressor and binds to a W-box element in the *TaNOX10* promoter to regulate reactive oxygen species (ROS) production and host resistance to *Pst.* CRISPR/Cas9 editing of the *TaWRKY19* gene yielded new wheat material with broad-spectrum resistance to *Pst* [71]. Blufensin1 (*Bln1*), serving as a susceptibility factor for a fundamental defense mechanism shared among cereal grain crops, including barley, wheat, rice and rye, interacts with the plasma membrane calmodulin *TaCaM3*, negatively regulating wheat stripe rust by disrupting Ca^2+^ influx [111]. *TaBln1* provides a new target for CRISPR editing to achieve durable resistance against *Pst*. *TaPsIPK1*, a wheat receptor-like cytoplasmic kinase gene, is also an *S* gene that is hijacked by the *Pst* effector PsSpg1. CRISPR/Cas9-mediated deletion mutants of *TaPsIPK1* have broad-spectrum resistance to *Pst* without negatively impacting other vital agronomic traits [72].

Rice blast is a devastating global fungal disease caused by *Magnaporthe oryzae*. Ethylene responsive factors (ERFs) belong to the superfamily AP2/ERF (APETALA2/Ethylene response element binding factors) and play a critical role in the defense response to *M. oryzae*. The *OsERF922* gene is reported to be an ERF transcription factor that negatively regulates blast resistance. Wang et al*.* employed CRISPR/Cas9 to silence the *OsERF922* gene, which significantly enhanced resistance to rice blast [74]. TGA-type transcription factors (TFs), which control the expression of defense genes, are key regulators of plant innate immunity. Niu et al*.* characterized a novel mechanism where the rice nucleus-localized casein kinase II (CK2) complex compromises transcriptional suppression of rice blast defense-related genes by phosphorylating OsTGA5 on Ser-32, a negative regulator of rice resistance against blast fungus and the closest rice homolog to *Arabidopsis TGA2*. Noteworthy, the *OsTGA5* knockout mutant created by CRISPR/Cas9 enhanced rice blast resistance without significantly affecting major agronomic traits [75]. In addition, lesion mimic mutants (LMMs) display a hypersensitive response-like phenotype, forming lesions of different sizes and shapes on tissues or organs such as leaves and sheaths through programmed cell death (PCD) in the absence of apparent stress or pathogens. Most LMM genes encode negative regulators of immunity by inducing the expression of defense genes to confer broad-spectrum disease resistance, making them ideal candidates for efficiently creating targeted genome edits to generate complete or partial loss-of-function alleles. Recently, Sha et al. isolated an LMM from a mutagenized rice population and identified a key gene, named *RESISTANCE TO BLAST1* (*RBL1*), which is responsible for conferring the LMM phenotype [79]. *RBL1* contains a 29-bp deletion and encodes a cytidine diphosphate diacylglycerol synthase (CDS) involved in phospholipid biosynthesis. The phosphatidylinositol derivative Ptdlns(4,5)P_2_ in rice is a susceptibility factor that plays an important role in effector secretion and fungal infection. Using genome editing, researchers obtained an allele of *RBL1*, named *RBL1*^Δ12^, which confers broad-spectrum disease resistance against three pathogens: *M. oryzae*, *Xanthomonas oryzae* pv. *oryzae* (*Xoo*), and *Ustilaginoidea virens* [79]*.* Importantly, this mutation did not decrease yield in small-scale field trials. Moreover, targeted knockouts of the *OsSEC3A*, *Pita*, *Pi21*, *bsr-d1* and *bsr-k1* genes were shown to confer varing degrees of resistance to *M. oryzae* [76–78]. CRISPR-mediated base editing has been used to reactivate the *Pi-d2* gene encoding RLK in rice, resulting in blast resistance [112].

Gray mold, caused by *Botrytis cinerea*, is a highly destructive fungal disease of numerous dicotyledonous crops. The prolonged use of succinate dehydrogenase inhibitor (SDHI) fungicides has led to resistance issues and pesticide contamination. Leisen et al*.* established a robust multiple-genome editing system of *B. cinerea* by utilizing co-transformation of fungal protoplasts, which combines optimized delivery of Cas9-sgRNA ribonucleoprotein complexes (RNPs) with transiently selected telomere vectors [113]. This system produced varying levels of resistance to SDHI fungicides by introducing a site-directed mutation at codon 272 of the *sdhB* gene. The RNP-based CRISPR/Cas genome-editing system with telomere vectors is effective in manipulating another devastating filamentous fungus, *M. oryzae*, which has the potential to be applied to other fungi; this approach can accelerate genetic modification in fungi, enabling in vivo structure‒function analysis of proteins and allowing for the study of fungicide resistance mechanisms [113]. Additionally, two necrotrophic pathogens, *Sclerotinia sclerotiorum* and *B. cinerea*, lead to stem rot and gray mold disease, respectively, posing a major threat to rapeseed (*Brassica napus*). Zhang et al. employed CRISPR/Cas9 to edit the *BnQCR8* gene, a conserved subunit of the cytochrome b-c1 complex in the plant respiratory chain that has eight homologous copies in the rapeseed cultivar Westar. By reducing the copies of *BnQCB8*, mutants with one or more edited copies showed strong resistance to *S. sclerotiorum* and *B. cinerea*, while no significant difference was observed in agronomic traits compared with wild type Westar [101]. Since the *QCR8* gene is widely conserved in plants, this research provides an important genetic resource for developing crop varieties with high resistance to these two pathogens.

Fusarium ear rot (FER) results in substantial yield losses of maize and mycotoxin contamination. *F. verticillioides* and *F. graminearum* cause Fusarium head blight (FHB) in wheat and maize stalk rot. *Fhb1* is a crucial quantitative trait locus (QTL) for enhancing FHB resistance, and knocking out the *TaHRC* allele susceptible to *Fhb1* could enhance FHB resistance [73]. As FER and FHB may be caused by the same pathogen, it is plausible to use the engineered *TaHRC* homolog in maize to cultivate resistance to FER. Liu et al*.* employed CRISPR/Cas9 to precisely edit the maize endogenous gene *ZmFER1*, creating null mutants that confer resistance to FER in maize across multiple environments [88]. Ma et al*.* revealed that both *ZmCOI1a* and *ZmJAZ15* participate in the jasmonate (JA) signaling pathway, which plays a vital role in maize immunity to *Gibberella* stalk rot (GSR, also known as maize stalk rot). The CRISPR/Cas9-mediated zmjaz15 loss-of-function mutant was more resistant to GSR [90]. *Ustilago maydis*, another major pathogen causing galls on all aerial parts of maize plants. Pathi et al*.* employed CRISPR/Cas9 to generate loss-of-function mutations in the *S* factor LIPOXYGENASE 3 gene (*LOX3*), largely reducing susceptibility due to ROS bursts during *U. maydis* infection [89]. In addition, Fusarium wilt, caused by *Fusarium oxysporum* f.sp. *niveum* (*FON*), is a severe vascular fungal disease that harms many crops, for which there is no effective resistant germplasm resource currently available. Studies have shown that phytothiokine (PSK) signaling can attenuate plant immune responses. Zhang et al*.* used CRISPR/Cas9 to knock out the *Clpsk1* gene encoding the PSK precursor, and resistance assessment showed that edited watermelon seedlings were more resistant to *FON* infection [102]. Moreover, microrchidia (MORC) family proteins, crucial nuclear regulators, are involved in epigenetic gene silencing and maintaining genome stability in both animal and plant species. Seven *MORC* genes (*AtMORC1-7*) and many homologs have been identified in various mono- and di-cotyledonous plants, especially *AtMORC1*, *AtMORC2* and *AtMORC6*, which regulate immune defense against a range of pathogens, including turnip crinkle virus (TCV), *Hyaloperonospora arabidopsidis*, and *Pseudomonas syringae*. It is noteworthy that each *AtMORC* family protein is species-specific. Recently, Galli et al*.* employed CRISPR/SpCas9 to double-knockout (dKO) barley genes *MORC1* and *MORC6a*, where the dKO *hvmorc1/6a* showed the strongest resistance to *Blumeria graminis* and *F. graminearum* [92].

### Resistance against Oomycete pathogens

Oomycetes, a vital group of eukaryotic microorganisms, produce highly resistant thick-walled oospores through sexual reproduction, playing a critical role in disease control and protection against pathogen invasion. However, the regulatory mechanism of oomycete reproduction remains unclear. CRISPR/Cas9-mediated editing of susceptible genes has been employed in crop breeding for disease resistance, but there have been relatively few reports in oomycetes. This section highlights the latest advancements in developing novel oomycete disease-resistant materials through gene editing and functional genetic studies. Soybean root rot, caused by *Phytophthora sojae* (*P. sojae*), leads to significant yield losses throughout the growth period. During the infection process, *P. sojae* secretes a large number of effector proteins targeting host factors. Genetically modifying these targets is a promising strategy for breeding disease-resistant soybean varieties. Liu et al*.* utilized CRISPR/Cas9 to deactivate the *GmTAP1* gene in soybean. It is known that the *P. sojae* effector PsAvh52 suppresses soybean immunity by targeting GmTAP1, increasing susceptibility to Phytophthora root rot. Consequently, three soybean plants with GmTAP1 functional deficiencies were cultivated, displaying enhanced resistance to *P. sojae* with minimal impact on the host plant's basal immunity. Furthermore, field trials demonstrated no agronomic penalties in these new soybean lines [99]. In addition, *Pythium ultimum*, one of the most destructive oomycetes, causes root rot or wilting in over 300 plant species. Feng et al. investigated PuM90 function, a developmental stage-specific Puf family RNA-binding protein that regulates sexual reproduction in *P. ultimum* by specifically binding to the 3´-UTR of its target mRNA, using CRISPR/Cas9-mediated gene knockout and in situ complementation methods [114]. *Phytophthora soiae* causes soybean root rot and provides a fitting model species for oomycete functional genomics research. Qiu et al*.* reported an in situ complementation method for precise restoration of a mutated gene within the CRISPR/Cas9-mediated gene knockout system for *Phytophthora*, which was used to confirm the biological function of a conserved regulatory B-subunit of protein phosphatase 2A (PsPP2Ab1) in the growth, sporulation and pathogenesis of *P. soiae* [115]. This study overcomes a technical challenge in the field of oomycete gene editing and will promote in-depth research on oomycete functional genomics.

### Resistance against bacterial pathogens

Bacterial blight caused by *Xanthomonas oryzae* pv. *oryzae* (*Xoo*) and bacterial leaf streak (BLS) caused by *X. oryzae* pv. *oryzicola* (*Xoc*) are two devastating rice diseases. During infection, *Xoo* utilizes the type III secretion system (T3SS) producing transcription activator-like effectors (TALEs) that function as eukaryotic transcription factors, which bind to effector-binding elements (EBEs) in the promoter region and use a TALE-encoded central repeat region (CRR) to induce transcriptional expression of *S* genes like *SWEET*, thereby establishing host susceptibility [80]. Identifying TALE-targeted genes in plants and modifying EBEs may represent an effective strategy to improve resistance against *Xoo* and *Xoc*. At least 20 target genes from the rice *SWEET* family have been identified, of which only *OsSWEET11*, *OsSWEET13*, and *OsSWEET14* are *Xoo S* genes. Multiplex editing was employed to target the promoter regions of these three genes using CRISPR/Cas9, yielding rice lines with broad-spectrum resistance to *Xoo* [80]. Furthermore, it is feasible to generate a loss-of-function* OsEDR1* mutant using Cas9 to edit the promoter region of the *Xa13* gene and induce site-directed mutations at the *IR24*, *Os8N3*, and *Xig1* genes. These interventions have improved the resistance of rice varieties to *Xoo* [81–85]. Few *S* genes have been reported for BLS (*Xoc*) compared with bacterial blight (*Xoo*). Xu et al*.* utilized CRISPR/Cas9 to induce mutations in the promoter region of the gene *OsSULTR3;6* (a TALE-targeted *S* gene for BLS), which encodes a predicted sulfate transporter in rice, creating a new germplasm exhibiting broad-spectrum resistance to *Xoc* [86]. Citrus canker, caused by *Xanthomonas citri* subsp. *citri* (*Xcc*), is a global highly destructive disease. Su et al*.* edited the canker susceptibility gene *CsLOB1* through transformation of embryogenic protoplasts with Cas12a/crRNA ribonucleoprotein, creating transgenic-free canker-resistant *Citrus sinensis* lines. These modified lines showed resistance to canker disease by effectively eliminating canker symptoms and inhibiting *Xcc* growth. It is noteworthy that the transgenic-free canker-resistant *C. sinensis* lines have received regulatory approval from USD APHIS and are not regulated by the EPA [103]. Moreover, the knocked-out citrus *S* gene *CsLOB1* and transcription factor *CsWRKY22* via CRISPR‒Cas9-mediated promoter editing conferred resistance to *Xcc* [104, 105]. Additionally, silenced tomato *Sijaz2* gene via CRISPR/Cas9 showed resistance to bacterial speck disease [95].

*DMR6* (*downy mildew resistance 6*), an *S* gene found in *A. thaliana*, is widely conserved among tomato, cacao and cassava. *Arabidopsis DMR6* encodes 2-oxoglutarate Fe(II)-dependent oxygenase (2OGO), which is upregulated during infection by oomycetes and bacteria. Both *AtDMR6* and its paralog, *AtDLO1* (*DMR6-Like Oxygenase1*), negatively regulate plant immunity and are co-expressed during pathogen infection. Tripathi et al*.* employed CRISPR/Cas9-mutated *MusaDMR6* orthologues, enhancing resistance to banana *Xanthomonas* wilt (BXW), one of the most destructive diseases affecting bananas caused by *Xanthomonas campestris* pv*. musacearum* (*Xcm*) [106]. Thomazella et al*.* characterized two *AtDMR6* orthologs in tomato, *SIDMR6-1* and *SIDMR6-2*. CRISPR/Cas-mediated *SIDMR6-1* mutants exhibited broad-spectrum resistance against *Phytophthora syringae*, *Phytophthora capsica* and *Xanthomonas* spp. [96]. Similarly, Kieu et al. employed CRISPR/Cas9 to inactivate the potato *StDMR6-1* gene and increased resistance against *Phytophthora infestans* [91].

### Exploring effective ways to engineer virus resistance

Eukaryotic translation initiation factors (elFs) are encoded by recessive anti-phytoviral genes, including elF4E, elF4G and other proteins. *eIF4E* is a vital susceptibility factor in plant‒virus interactions. Impaired functional domains of latent *eIF4E* and its isoform, *eIF(iso)4E,* can initiate immune responses in many potyviruses. Chandrasekaran et al*.* used CRISPR/spCas9 to knockout the recessive *eIF4E* gene in cucumber, and homozygous T3 generation plants showed immunity to cucumber vein yellowing virus (CVYV) and resistance to two potyviruses, zucchini yellow mosaic virus (ZYMV) and papaya ringspot mosaic virus-W (PRSMV-W) [107]. CRISPR/spCas9-mediated *eIF4E*-edited cassava has conferred resistance to cassava brown streak disease caused by cassava brown streak virus and Ugandan cassava brown streak virus [108]. Similarly, editing *eIF(iso)4E* in *A. thaliana* enhanced resistance to turnip mosaic virus (TuMV), while the *eIF4G* knockout mutation in rice strains conferred resistance to rice tungro spherical virus (RTSV) [87, 116]. Furthermore, Bastet et al*.* employed the CRISPR/nCas9-mediated cytidine deaminase editing system to introduce a C-to-G single-point mutation (N176K) and convert *eIF4E1* into a resistance allele in *A. thaliana*, conferring resistance to clover yellow vein virus (CIYVV) [117]. Site-specific mutagenesis of the tomato *eIF4E1* gene and *PDIL5-1* (protein disulfide-isomerase-like 5–1 gene) has also boosted resistance to pepper mottle virus (PepMoV) and barely mild mosaic virus (BaMMV), respectively [93, 97]. In a study, CRISPR/Cas9 was used to edit the *ACT* gene of African cassava mosaic virus (ACMV), a member of a widespread and important family (*Geminiviridae*) of plant‒pathogenic DNA viruses. However, the edited materials did not show resistance to the virus during glasshouse inoculations. Sequence analysis revealed that 33% to 48% of the edited virus genomes had evolved a conservative single nucleotide mutation at the target recognition site, avoiding CRISPR/Cas9 cleavage [118]. The authors argued that the use of CRISPR/Cas accelerated the evolution of geminiviruses, posing a potential biosecurity risk [118]. Although this research has certain limitations, it highlights the importance of strict review and cautious implementation of technologies with the potential to accelerate virus evolution to prevent significant biosafety risks [119]. Most plant disease resistance genes (*R*) encode nucleotide binding-leucine-rich repeat (NLR) proteins. The TIR-NLR gene *Prv*, identified in melon, is a candidate gene linked to resistance against papaya ringspot virus (PRSV). Nizan et al. employed CRISPR/Cas9 to mutate the melon *Prv* gene, demonstrating its pivotal role in conferring resistance to PRSV infection. Intriguingly, one of the *Prv* mutant alleles not only disrupted the resistance effect but also led to a severe dwarfing phenotype, accompanied by a temperature-dependent defense response with elevated SA levels. This study represents the first successful application of CRISPR/Cas9 to confirm the function of an *R* gene in melon [120].

### CRISPR/Cas-mediated insect-resistance engineering

Transgenic *Bt* cotton and *Bt* maize have been effective in controlling lepidopteran pests such as cotton bollworm (*Helicoverpa armigera*) and Asian corn borer (*Ostrnia furnacalis*). However, *Bt* crops are ineffective in resistance to other pests like aphids, and encounter resistance from regulatory agencies and social critics due to the random insertion of exogenous genes. Genome editing provides a green, cost-effective way to cultivate insect-resistant crops. Currently, relatively few reports exist about CRISPR/Cas-mediated insect genome editing for integrated pest management. Lu et al*.* employed CRISPR/Cas9 to inactivate the *OsCYP71AI* gene in rice, disrupting serotonin biosynthesis through higher concentrations of salicylic acid, conferring resistance to rice brown planthopper (BPH, *Nilaparvata lugens*) and striped stem borer (SSB, *Chilo suppressalis*) [121]. Guo et al*.* demonstrated that 4-vinylanisole (4VA) is an aggregation pheromone for the migratory locust (*Locusta migratoria*). Knocking out the *OR35* gene (coding for a specific olfactory receptor for 4VA) using CRISPR/Cas9 significantly reduced the antennae electrophysiological responses and weakened the attractiveness of behavior 4VA [122]. This study provides a new strategy for monitoring and managing locust plague. Moreover, Li et al*.* applied CRISPR/Cas9 to knockout the *Bactrocera dorsalis* *NPFR* gene, creating *BdsNPFR*^−/−^ mutants that significantly downregulated the transcriptional levels of several olfactory receptor genes, including *Orco*, and reduced electrophysiological responses to various odorants while markedly prolonging foraging time and decreasing the foraging success rate [123]. This study enriched the molecular regulatory mechanism of foraging behavior in *B. dorsalis* and established a theoretical foundation for the development of behavioral control agents targeting the olfactory system.

### CRISPR/Cas-mediated herbicide-resistance engineering

Acetolactate synthase (ALS) is a pivotal enzyme in branched-chain amino acid biosynthesis, where targeted point mutations can confer herbicide resistance to sulfonylurea (SU) and imidazolinone (IMI), allowing successful weed management in crops, including rice, maize, wheat and cotton. Shimatani et al*.* used a cytidine-base multiplex editing (CBEs) system to introduce specific base transitions in the *OsALS* gene, developing numerous herbicide-resistant rice lines while preserving *ALS* activity [51]. Kuang et al*.* modified the *OsALS1* gene via a base-editing-mediated gene evolution (BEMGE) approach, generating plant lines containing the P171F mutation with varying degrees of tolerance to the herbicide bispyribac-sodium [124]. Similarly, Zhang et al. employed CBEs to create abundant missense mutations in the *OsALS* gene and found that four different mutations at the P171 codon exhibited varying ALS-inhibiting tolerance [125]. Wu et al*.* optimized CBE to mutate the *BnALS1* gene at position P197 in oilseed rape (*Brassica napus*), conferring resistance to tribenuron-methyl [126]. Jiang et al*.* developed herbicide-resistant maize germplasms harboring the P165S mutation or W542L/S621I double mutations in *ZmALS1* and *ZmALS2* using prime editing that optimized pegRNA expression [127]. Acetyl-coenzyme A carboxylase (ACCase), a pivotal enzyme in lipid biosynthesis, serves as a primary target for modulating herbicide interactions. By employing targeted saturation mutagenesis on specific amino acids within the carboxyltransferase (CT) domain of the ACCase gene, the direct modulation of its herbicide interaction can be achieved. Li et al*.* developed novel saturated targeted endogenous mutagenesis editors (STEMEs) and identified four herbicide resistance mutation sites by near-saturated mutated and directed evolution of the *OsACC* gene in rice, among which P1927F and W2125C showed strong herbicide resistance [128]. Zhang et al*.* generated new wheat germplasms that are tolerant to SU, IMI and aryloxyphenoxy propionate-type multiple herbicides by base editing of *TaALS* and *TaACCase* genes [125]. Furthermore, pladienolide B (PB) and herboxidiene (GEX1A) represent two notable polyketide natural products from *Streptomyces* sp., which function as potent plant splicing inhibitors. SF3B1, a crucial splicing factor within the SF3B complex of the spliceosome U2 snRNP, has garnered considerable attention due to its remarkable efficacy as an herbicide. Butt et al*.* utilized a CRISPR/Cas-based directed evolution platform (CDE) to introduce mutations into the conserved structural domain HR15-17 of *OsSF3B1*, obtaining 21 edited rice strains that confer variable levels of resistance to GEX1A [129]. In addition, specific editing of the *EPSPS*, protoporphyrinogen oxidase (*PPO)* and α-tubulin homologue (*TubA2*) genes has been reported to confer resistance to glyphosate, butafenacil, trifluralin and herboxidiene (GEX1A), respectively [130–132]. Importantly, these herbicide-resistant alleles can serve as selectable markers for more gene editing events.

## Global regulation of genome-edited crops

A global regulatory framework that is both just and clear is fundamental to the future of genome editing technology [133]. Currently, the regulations for GEOs are inconsistent, with two main approaches based on product or process referencing GMOs. The US authorities categorize CRISPR/Cas-edited plants that lack foreign or recombinant DNA as non-regulated, allowing for their commercialization. This encompasses edited variants like bristlegrass, mushroom, canola oil from herbicide-tolerant Canola plant, starch from waxy maize, and high oleic acid soybean oil from Calyno soybean [133, 134]. Canada recently approved oligonucleotide-directed mutagenesis (ODM)-mediated canola [135]. Authorities in Argentina, Colombia, Chile, and Brazil are facilitating the development and deregulation of CRISPR/Cas-edited crops, considering them indistinguishable from natural mutations [135]. Meanwhile, the EU, Australia and New Zealand define organisms with CRISPR/Cas-mediated directed or random mutagenesis as GMOs, requiring strict regulations and precautions during commercialization [133, 136, 137]. Other countries in Latin America, India, and most less developed countries worldwide have not yet established regulations for evaluating CRISPR/Cas-edited plants [135]. Recently, China issued its first safety certificate for a gene-edited organism, i.e., the soybean variant AE15-18–1. This particular soybean variety has been genetically modified in the genes *gmfad2-1a* and *gmfad2-1b*, improving quality traits that are beneficial for production purposes. Furthermore, the publication of the Regulations for the Evaluation of Gene-edited Plants for Agricultural Use (Trial) has further accelerated the implementation of gene editing in breeding practices (www.moa.gov.cn). In conclusion, it is crucial to establish a comprehensive regulatory framework and risk assessment system for distinguishing gene-edited organisms from genetically modified organisms. This framework should build upon existing regulations and scientific risk assessment practices for traditional breeding and genetic engineering, regularly being updated to align with technological advancements and industrial applications. These measures are essential for unlocking the transformative potential of gene editing in driving sustainable development in modern agriculture.

## Bottlenecks and perspectives

The CRISPR/Cas system is an efficient and versatile tool for editing plant genomes, which has been used for over a decade to modify plant genomes for studying specific genes and biosynthetic pathways, as well as accelerating the breeding of numerous plant species, including both model and non-model crops [138]. However, the practical application of CRISPR/Cas9 still faces several challenges, such as PAM restrictions, limited editing target ranges, off-target effects, and defects associated with nuclease-induced DSBs. This section introduces the recent advances that help address these challenges, including the engineering and discovery of novel CRISPR/Cas systems with improved functionalities, vector optimization for Cas nucleases and gRNA delivery, and the development of DSB-free genome editors.

Successful in vivo gene editing with CRISPR/Cas hinges on the effective delivery of gene editing tools. CRISPR/Cas-based delivery systems primarily fall into two major categories: adeno-associated viruses and lipid nanoparticles. Researchers have developed a variety of innovative delivery vehicles utilizing ribonucleoprotein (RNP) complexes, engineered viral vectors (such as tobacco mosaic virus, foxtail mosaic virus, and Sonchus yellow net virus), as well as nanomaterials like carbon nanotubes, DNA nanostructures, and cell-penetrating peptides [139]. However, the current coding sequence lengths of CRISPR/Cas nucleases and related molecules exceed the capacity of most plant viral vectors (1–2 kb). The RNA virus tomato spotted wilt virus (TSWV) has a host range of over 1000 plant species. Liu et al*.* introduced a transient delivery system based on TSWV, which can effectively deliver large nucleases (eg. CRISPR/Cas12a and Cas9) as well as adenine and cytidine base editors to various host crop varieties [140]. Moreover, commonly used virus delivery vehicles can lead to sustained Cas9/sgRNA exposure in cells, resulting in genetic toxicity, extensive deletions, translocations, chromosomal segregation, and unwanted gene integrations. Additionally, base editors (BE) and prime editors (PE) that do not rely on DSBs and utilize HDR require larger packaging capacity. The engineered lentiviral-derived nanoparticles (LVNPs) developed by Haldrup et al. are conducive to the effective and safe delivery of CRISPR/Cas ribonucleoprotein complexes, thereby meeting the needs for base editing, prime editing, and in vivo gene modification applications [141].

The dependence on genotype for in vitro regeneration capacity undoubtedly remains a significant obstacle in the application of the CRISPR/Cas system. Additionally, the conventional approach to plant genetic transformation involving callus induction and regeneration is time-consuming and inefficient. To overcome these challenges, Cao et al. introduced the cut-dip-budding (CDB) delivery system, which utilizes *Agrobacterium rhizogenes* to directly inoculate explants, leading to transformed roots that produce transformed buds through root suckering, bypassing the need for traditional tissue culture [142]. This approach has successfully achieved heritable transformation or gene editing in various plant species, such as sweet potato, Paulownia, and dandelion [142]. Furthermore, the induction of shoot apical meristem in plants is controlled by key developmental regulators (DRs). By expressing specific DRs in leaf cells, it is possible to redirect cell development, induce meristematic tissue, and promote the conversion into other cell types. Maher et al*.* demonstrated the co-expression of DRs and gene editing elements in plants, resulting in the direct generation of stable genetically modified and gene-edited plants without reliance on tissue culture [143].

Leveraging growing genomic databases, metagenomics, protein engineering, and AI-assisted tools will significantly accelerate and broaden the application of novel gene editing technologies in therapeutics and agriculture through protein structure prediction [138]. Huang et al*.* utilized AI-assisted AlphaFold2 to pioneer the prediction and classification of precise genome-editing enzymes, such as ssDNA deaminases and dsDNA deaminases [144]. Furthermore, overcoming the size restriction of Cas enzymes will be crucial for maximizing the efficiency of these technologies. For the continuous screening of novel Cas enzymes, compact Cas enzymes with potential applications are essential. Developing activity-enhanced variants based on existing Cas systems is necessary to overcome PAM limitations and expand editing sites. Addressing off-target effects remains critical and ugent tasks. Previous studies have focused on improving editing efficiency by modifying promoters, utilizing tissue-specific promoters, and optimizing Cas codons. Recently, Hu et al*.* developed a modular base editing system (CyDENT) that performs CRISPR-free, strand-selective DNA editing [145]. CyDENT achieves efficient cytosine base editing in the cell nucleus, mitochondria, and chloroplasts, displaying significant strand specificity and low sequence preference in mitochondrial editing. With its wide genome-targeting capabilities, CyDENT provides a highly precise and broadly applicable base editing tool. These advancements in precise editing strategies for the nucleus and organelles in plant cells hold significant potential for applications in disease management and precision molecular breeding in agriculture [145].

## Conclusions

Improving crop varieties using the CRISPR/Cas system to resist biotic and abiotic stresses poses a formidable challenge, given that the majority of genetically improved plants are currently in the early stages of research. The intricate interplay of environmental factors in agricultural settings underscores the need for rigorous field trials under varying disease pressures and favorable conditions to fully exploit the practical advantages of these enhanced crops. Nevertheless, CRISPR/Cas-mediated genome editing represents a groundbreaking leap forward in agro-biotechnology, facilitating the precise and efficient introduction of mutations that contribute to crop enhancement and novel protective strategies. Furthermore, the collaborative advancement of this cutting-edge technology with high-throughput phenotyping, genomic selection, accelerated breeding methods, and synthetic biology is reshaping the landscape of agriculture with unparalleled impact. We anticipate that the establishment of a product-focused regulatory framework for gene editing could effectively balance safety considerations for human beings and the environment with the innovative potential of genome editing-based next-generation breeding. This will empower global farmers and consumers to harness the potential of this technology for sustainable agricultural practices.

## Data Availability

All data used in this study are included in this article.
